# Supplementary material for: Multivariate chemogenomic screening prioritizes new macrofilaricidal leads
Source: Commun Biol. 2023 Jan 13;6:44. doi: 10.1038/s42003-023-04435-8 (PMC9839782; doi:10.1038/s42003-023-04435-8)
Supplement: Supplementary file 1 — Supplementary Information [file 42003_2023_4435_MOESM1_ESM.pdf]

type	n
Adult only	846
All	10001
Female and MF	501
Female only	444
Male and MF	155
Male only	886
MF only	213
None	1750

Supplementary Table 1 - Comparison of *Brugia malayi* adult and microfilaria RNA-seq profiles[7]. RNA-seq data was acquired from NCBI's SRA database. Values for transcripts per million (TPM) were averaged across replicates and any gene with an average TPM of greater than 5 was considered to be "expressed."

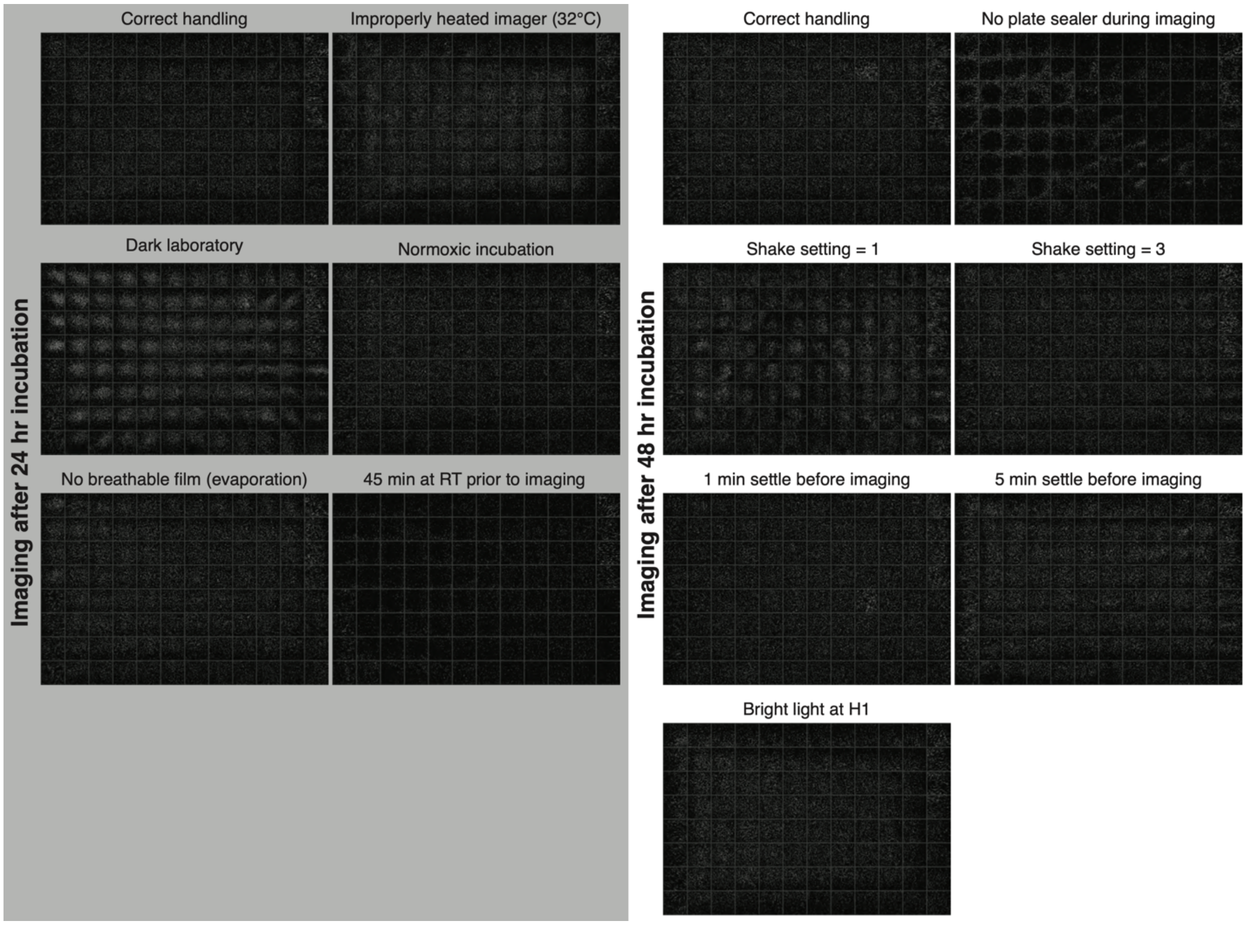

Supplementary Figure 1 - Assessment of microfilaria congregation in response to variations in handling and environment

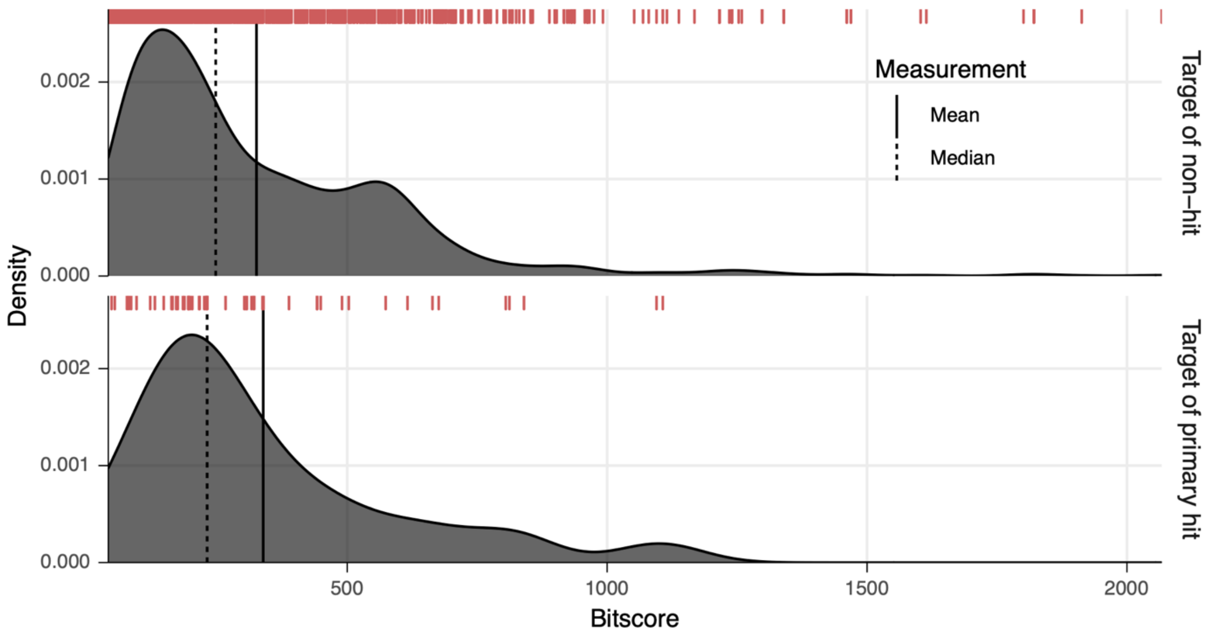

Supplementary Figure 2 - BLAST comparisons of the targets of hit and non-hit compounds

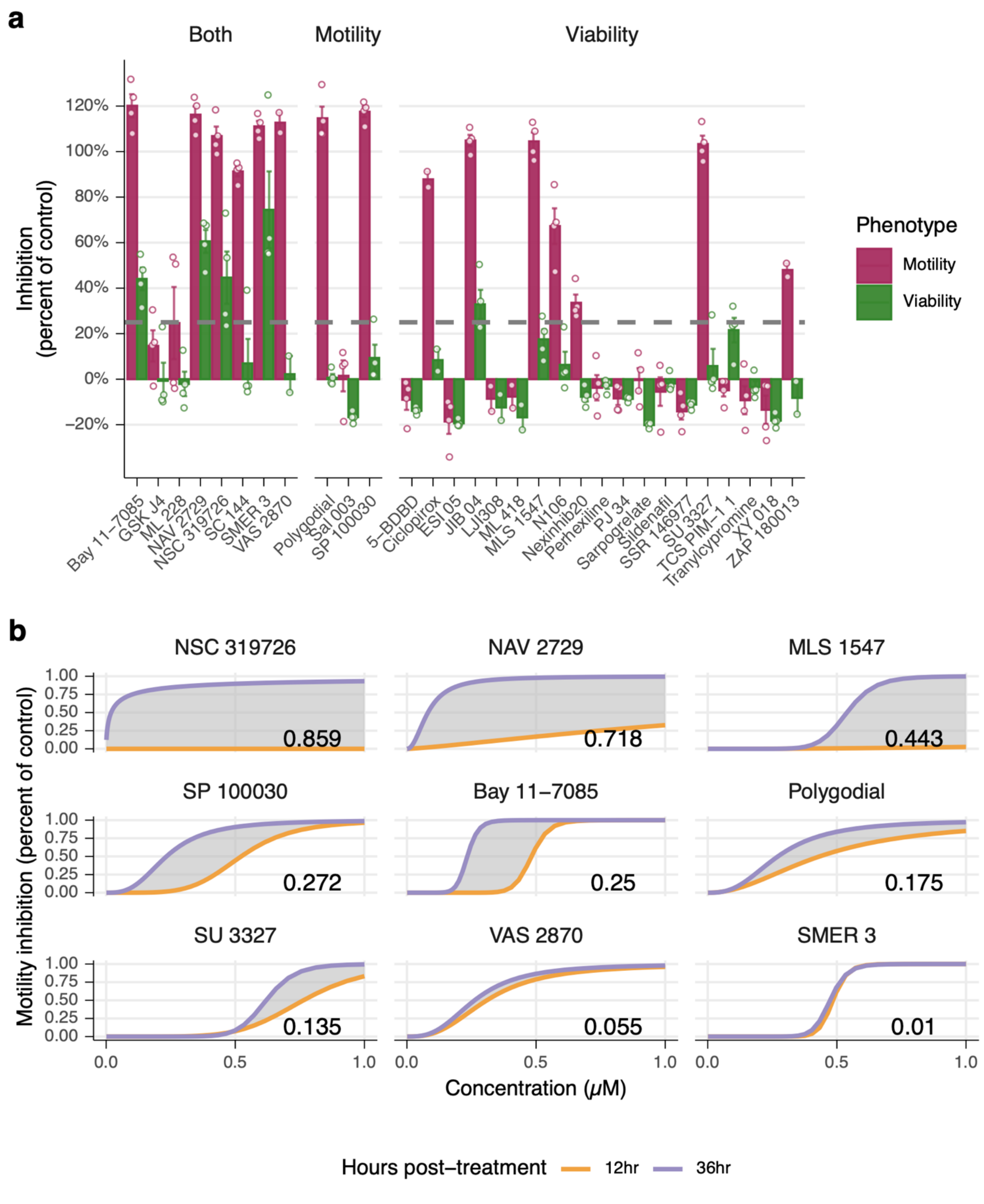

Supplementary Figure 3 - Dose-responses and pharmacodynamics of hit compounds. (a) The percent inhibition of each compound at 1 μM in the dose-response experiment. Error bars represent the standard error of the mean. (b) Dose-response curves from the same experiment

at 12- and 36-hours post-treatment. Black numbers in each facet indicate the area between the curves.

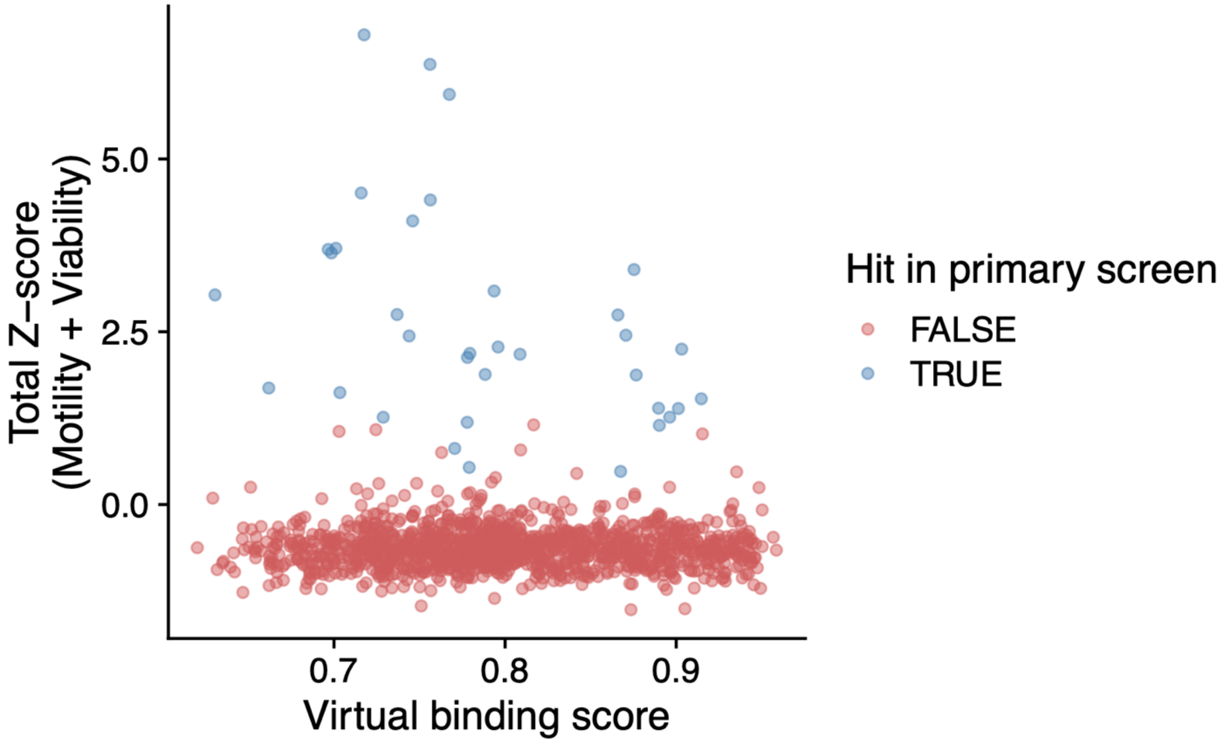

Supplementary Figure 4 - Correlation between docking probability and total Z-score from the primary screen.

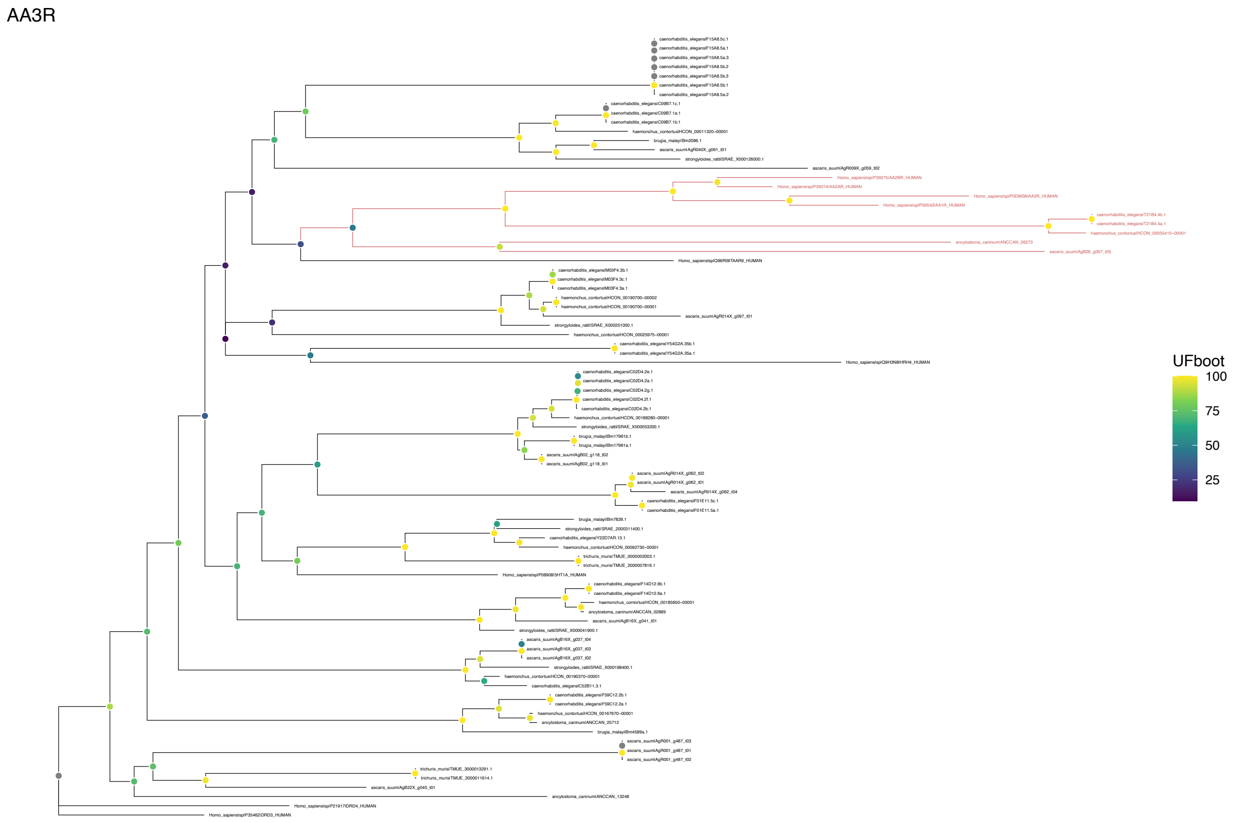

Supplementary Figure 5 – Phylogenetic tree of AA3R orthologs

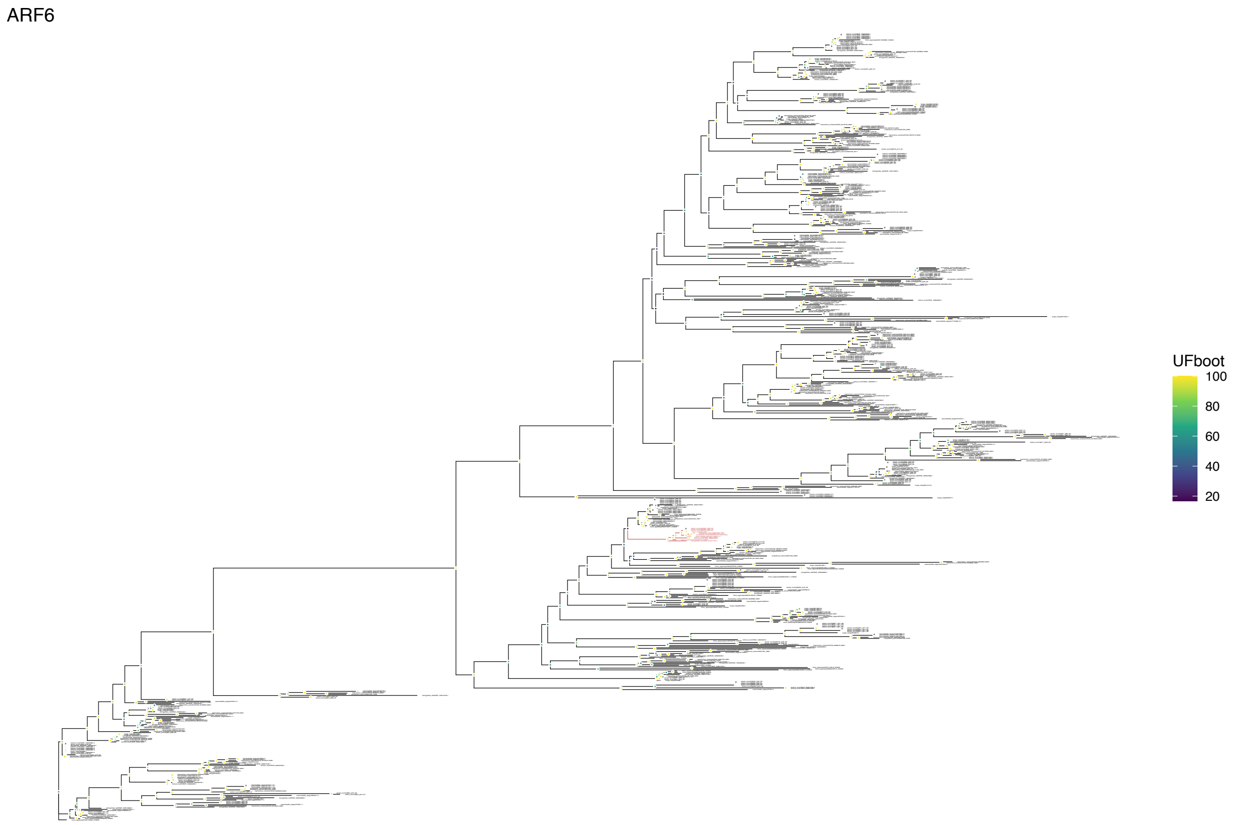

Supplementary Figure 6 – Phylogenetic tree of ARF6 orthologs

5HT2A

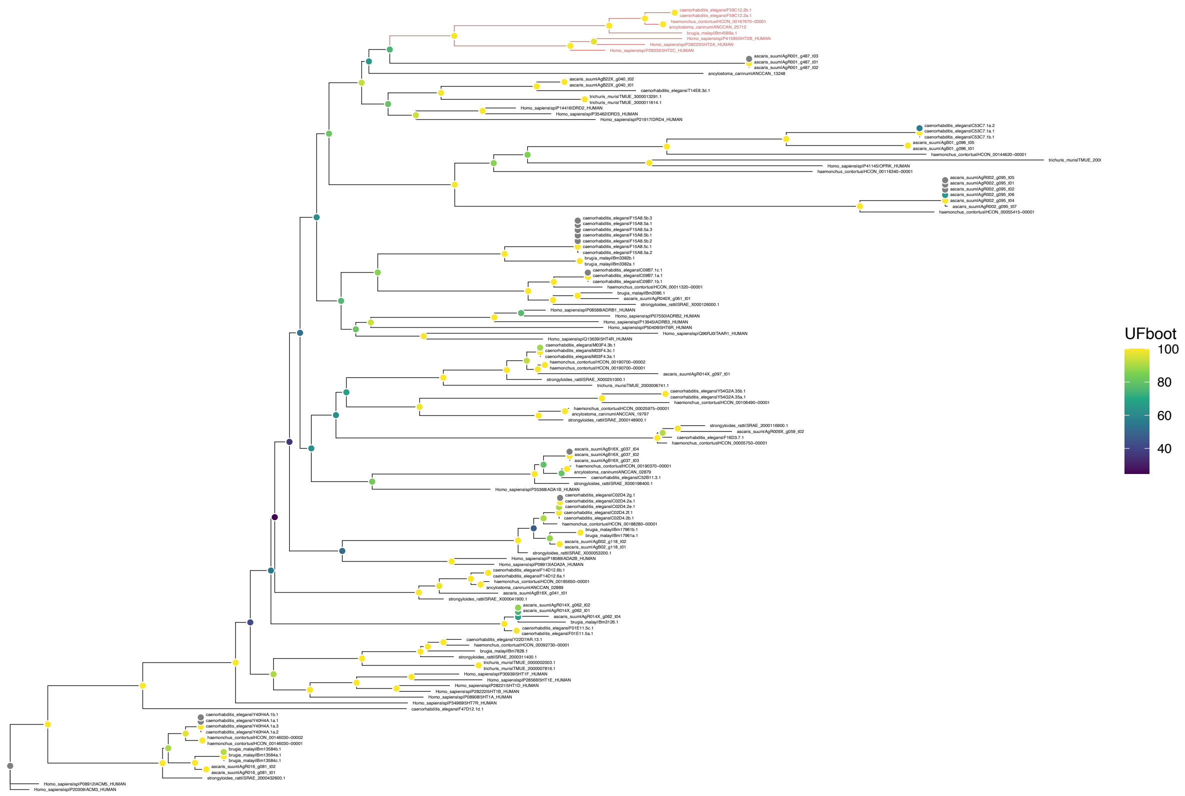

Supplementary Figure 7 – Phylogenetic tree of 5HT2A orthologs

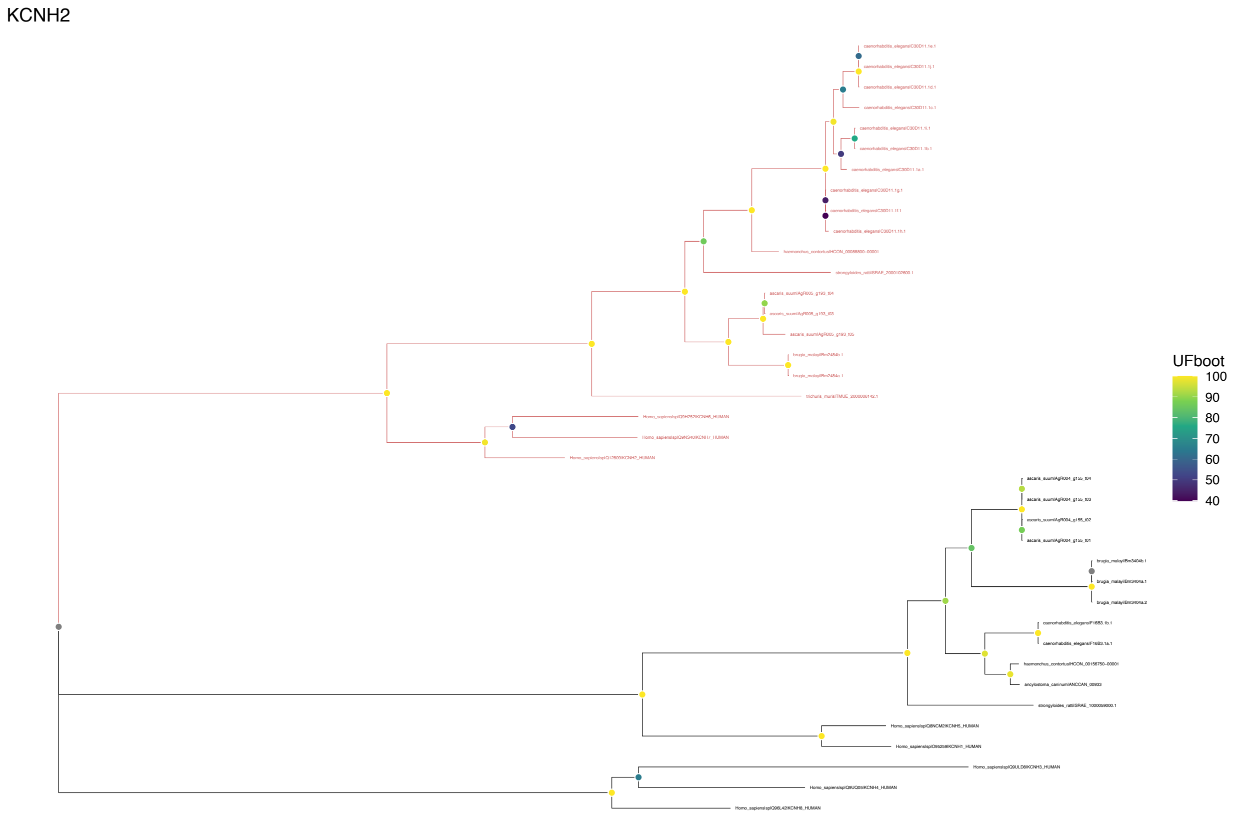

Supplementary Figure 8 – Phylogenetic tree of KCNH2 orthologs

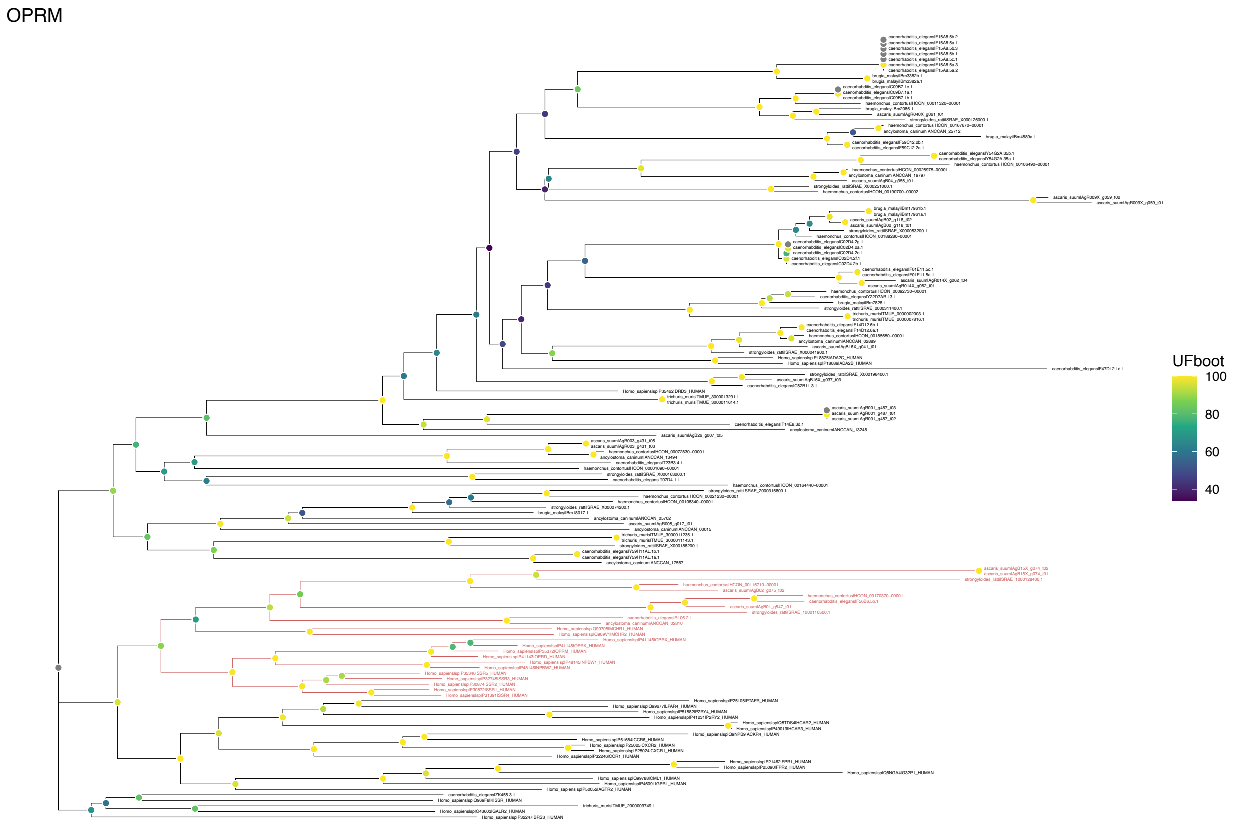

Supplementary Figure 9 – Phylogenetic tree of OPRM orthologs

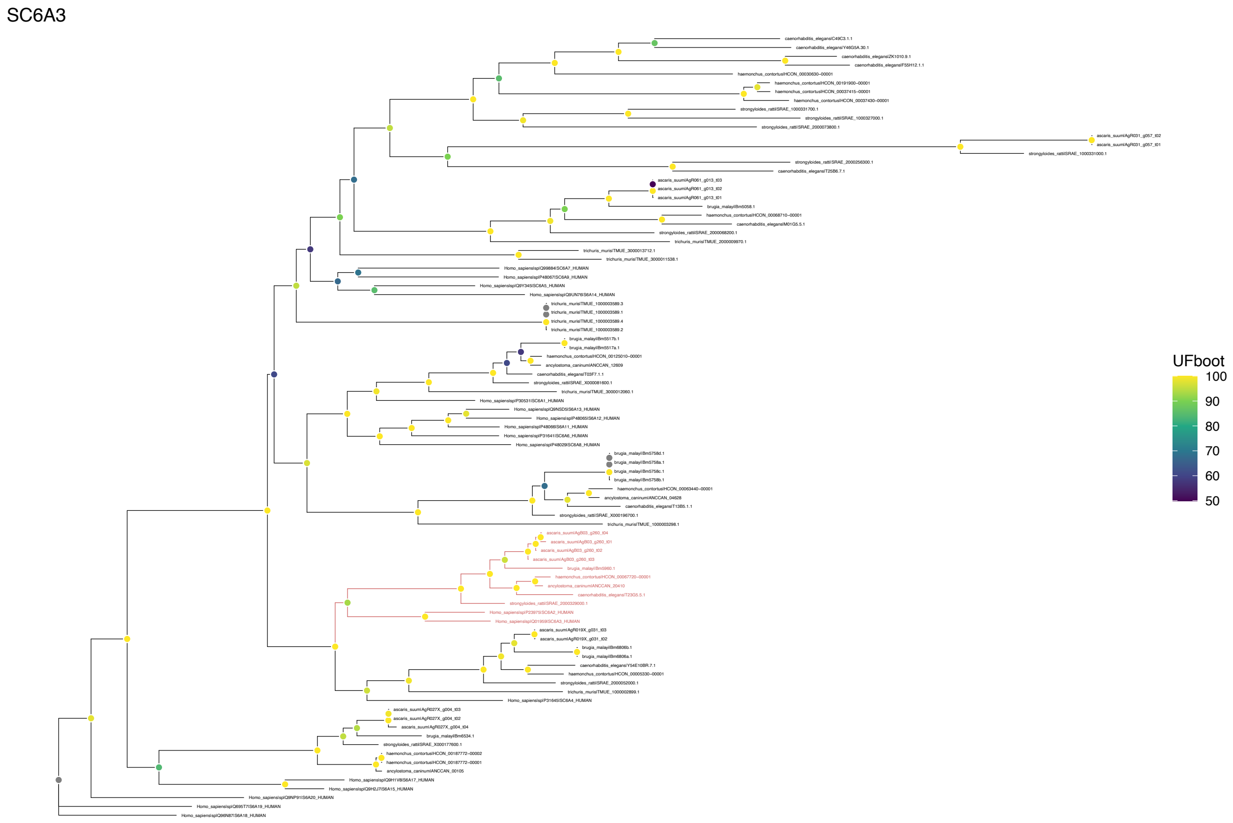

Supplementary Figure 10 – Phylogenetic tree of SC6A3 orthologs

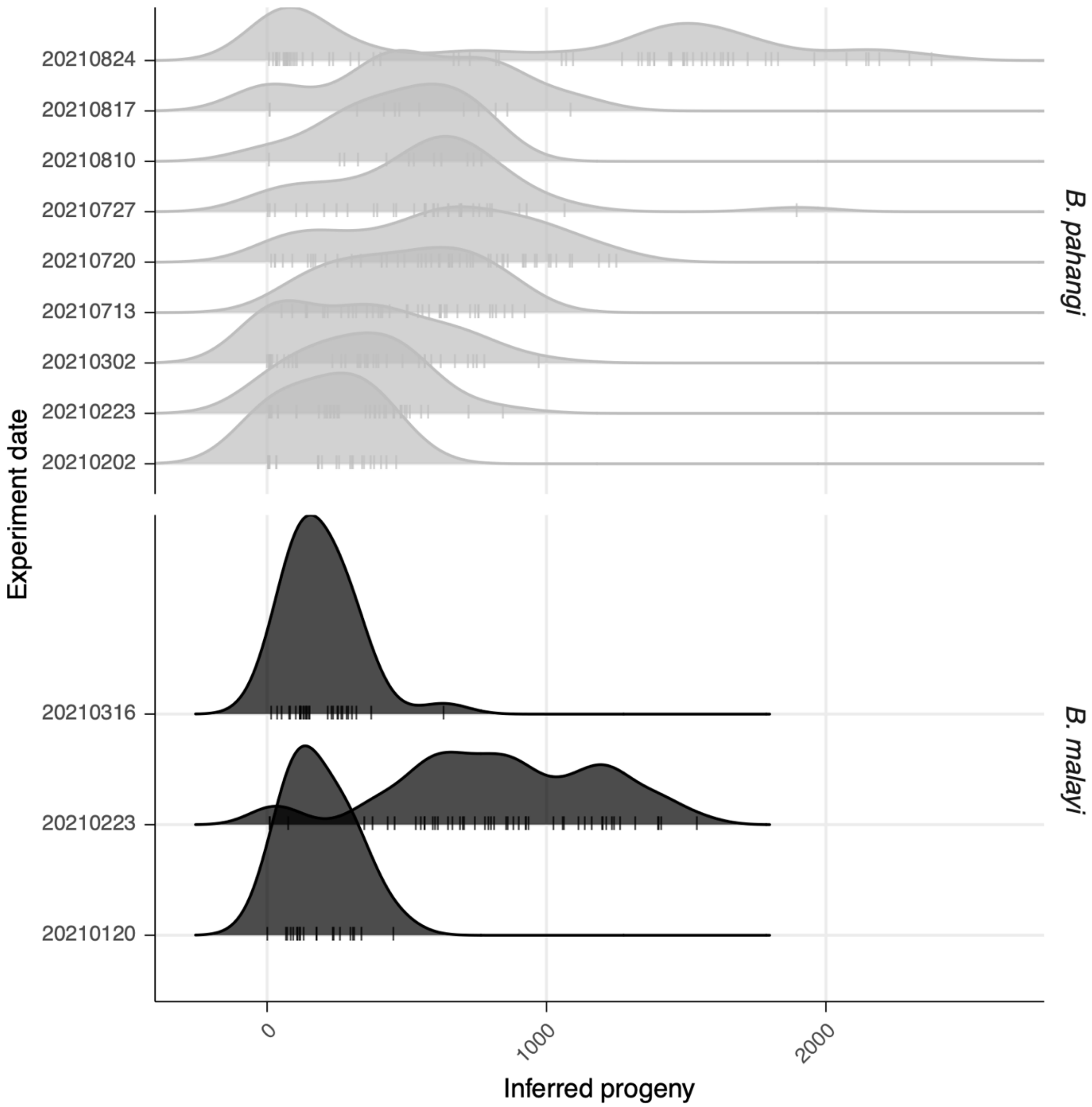

Supplementary Figure 11 - Batch variability of *Brugia* spp. fecundity

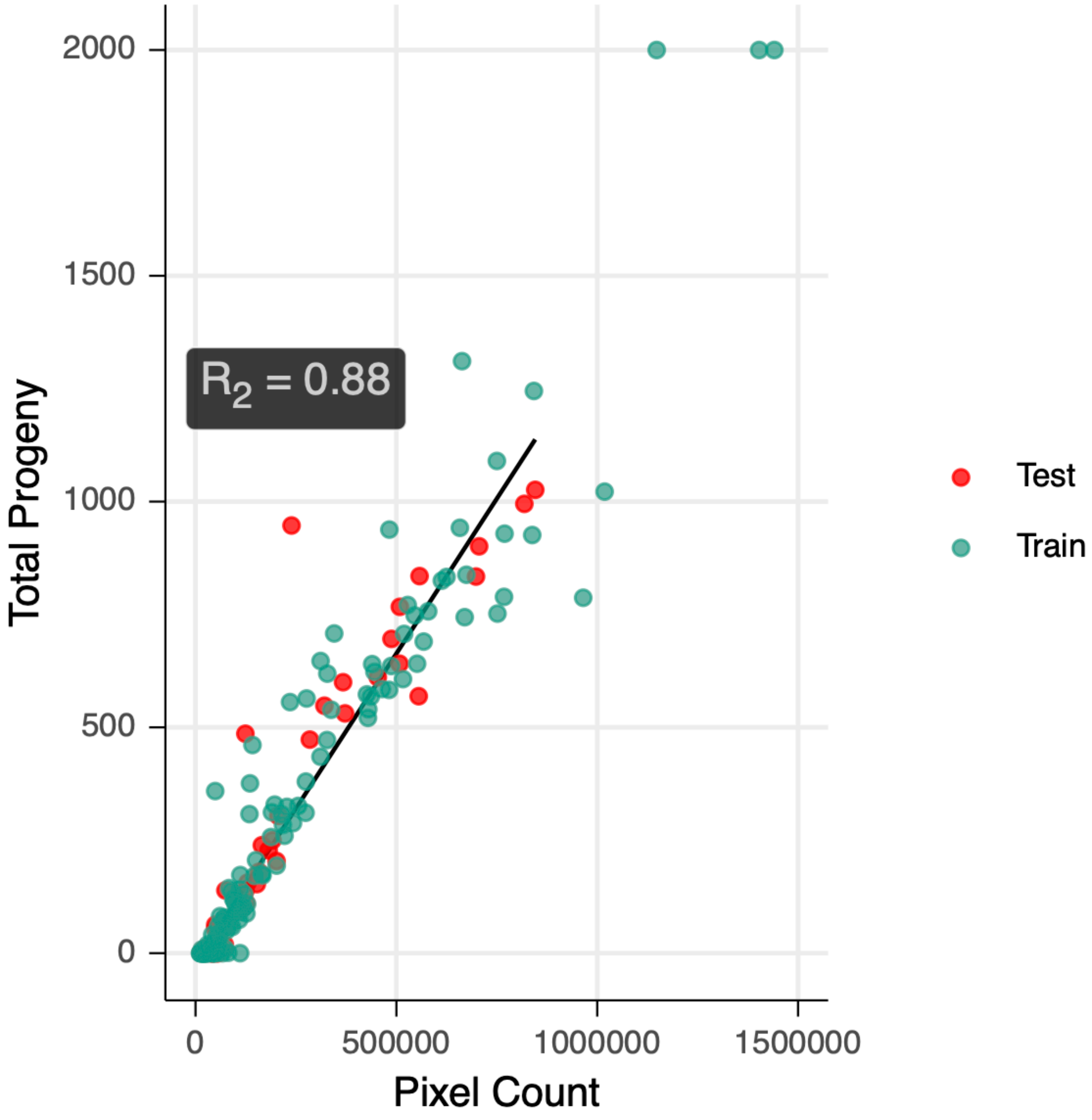

Supplementary Figure 12 - Evaluation of a linear model for estimating the number of microfilaria in a well
