# Supplementary material for: Multivariate chemogenomic screening prioritizes new macrofilaricidal leads
Source: Commun Biol. 2023 Jan 13;6:44. doi: 10.1038/s42003-023-04435-8 (PMC9839782; doi:10.1038/s42003-023-04435-8)
Supplement: Supplementary file 2 — Description of Additional Supplementary Files [file 42003_2023_4435_MOESM2_ESM.pdf]

Description of Additional Supplementary Files

File name: Supplementary Data 1

Description: Phylogenetic trees that include the human target for all hit compounds along with homologs from selected nematode species

File name: Supplementary Data 2

Description: A compilation of all the screening data for the hit compounds

File name: Supplementary Data 3

Description: List of the B. malayi target structure that were estimated by docking to be the most likely target for hit compounds

File Supplementary Data 4

Description: Tocriscreen 2.0 compounds in sanitized SMILES format used for molecular docking
